# BATVI: Fast, sensitive and accurate detection of virus integrations

**DOI:** 10.1186/s12859-017-1470-x

**Published:** 2017-03-14

**Authors:** Chandana Tennakoon, Wing Kin Sung

**Affiliations:** 1 Department of Computational and Systems Biology, Genome Institute of Singapore, Singapore, 138672 Singapore; 2 Department of Computer Science, National Unversity of Singapore, Singapore, 117417 Singapore; 3 UAE University, PO Box, 17551, Al Ain, United Arab Emirates

**Keywords:** NGS, Viral integration, Alignment

## Abstract

**Background:**

The study of virus integrations in human genome is important since virus integrations were shown to be associated with diseases. In the literature, few methods have been proposed that predict virus integrations using next generation sequencing datasets. Although they work, they are slow and are not very sensitive.

**Results and discussion:**

This paper introduces a new method BatVI to predict viral integrations. Our method uses a fast screening method to filter out chimeric reads containing possible viral integrations. Next, sensitive alignments of these candidate chimeric reads are called by BLAST. Chimeric reads that are co-localized in the human genome are clustered. Finally, by assembling the chimeric reads in each cluster, high confident virus integration sites are extracted.

**Conclusion:**

We compared the performance of BatVI with existing methods VirusFinder and VirusSeq using both simulated and real-life datasets of liver cancer patients. BatVI ran an order of magnitude faster and was able to predict almost twice the number of true positives compared to other methods while maintaining a false positive rate less than 1%. For the liver cancer datasets, BatVI uncovered novel integrations to two important genes TERT and MLL4, which were missed by previous studies. Through gene expression data, we verified the correctness of these additional integrations.

BatVI can be downloaded from http://biogpu.ddns.comp.nus.edu.sg/~ksung/batvi/index.html.

## Background

The discovery that Rous sarcoma virus can induce cancer in chicken [1] had revolutionized cancer research. Although this discovery was met with initial skepticism, the association between cancers and viral infections has been firmly established today. One of the striking facts is that some viruses causing cancers are highly prevalent. For example, the Epstein-Barr virus infects about 90% of the human population by adolescence and it causes several forms of cancer [2]. Another example is Hepatocellular carcinoma (HCC). Its leading cause is Hepatitis B virus (HBV) infection. At least one third of the world population has been infected by HBV at some point in their lives [3]. Therefore, the study of the relationship between virus integrations and diseases is very important.

The revolution of next generation sequencing (NGS) enables us to probe the interactions of viral and human DNAs at a molecular level. Apart from detecting the presence of viruses, NGS enables the detection of the virus integrations and determination of the actual integration breakpoints at base-pair resolution. However, this analysis is not straightforward. One reason is that the majority of the NGS reads are originated from the host (human) instead of the virus. Another reason is that the integrations might happen in repeat regions, and may produce ambiguous alignments. Therefore, it is important to find a method that can effectively use a small number of reads to predict the presence of viral integrations.

Several methods were proposed to study the presence of viruses and their integrations. SRSA [4] and PathSeq [5] are some early programs that were designed to determine the types of viruses and pathogens in NGS samples. Recently, VirusSeq [6], ViralFusionSeq [7] and VirusFinder [8, 9] were proposed to identify virus integration sites. VirusSeq first aligns reads to a reference genome; then, the unaligned reads are mapped to a virus database to identify the target viral genome. This viral genome is added as a dummy chromosome and all unmapped paired-end reads are realigned to this modified genome. By identifying discordant alignments between human and the viral genome, integration sites are identified. In ViralFusionSeq, the reads are aligned to viral and human genomes using BWA-SW [10]. Reads having soft clips in the viral mappings, and reads having one end aligned to human genome while the other end aligned to virus genome are extracted. These reads are clustered and assembled (if possible) to find integrations. VirusFinder aligns reads to both human and viral genomes. Then, virus integrations are found by the structural variation callers SVdetect [11] and CREST [12].

We identify several drawbacks with existing approaches. First, existing methods use general NGS read aligners to identify reads near the virus integration sites. General NGS real aligners assume each read contains some long seed with low number of mismatches. However, such assumption may not be valid on the virus genome (which have high mutation rate) or near to the virus integration sites. Together with the fact that the number of reads covering virus integrations are usually lower, existing methods has difficulty to align reads around virus integration sites, which reduce the sensitivity of existing methods to predict virus integrations. The second problem is the use of soft-clipped reads by ViralFusionSeq and VirusFinder. The soft-clip positions predicted by the aligners may not be accurate. Finally, VirusSeq and VirusFinder assume that exactly one virus strain is involved in the integrations. (Note that VirusFinder 2 provides an option to choose the virus reference). However, there are cases where a single individual is infected by multiple different strains of the same virus [13]. Therefore, these methods may fail to call some integration sites.

In this paper, we propose a method BatVI to overcome these problems. BatVI identifies a set of probable chimeric reads using the sensitive BLAST aligner [14]. BLAST is able to detect chimeric reads with short viral segments (as small as 18 bp) accurately. Therefore, BatVI can detect viral integrations having very low coverage. For detecting viral integrations, BatVI uses fast clustering and multiple sequence assembly methods. Furthermore, BatVI does not make any assumption about the strains of the integrated virus.

We compare BatVI with VirusFinder 2 and VirusSeq. (We did not include ViralFusionSeq as it did not finish in the allocated time). Using simulated data, we show that BatVI recovers more viral integrations. Furthermore, we note that existing methods may predict many false integrations that occur in repeat regions. On the other hand, BatVI can either identify the correct integration or report the fact that the integration is unreliable. We also test the performance of BatVI using real datasets. Using a list of viral integrations generated by high coverage target sequencing [15] as a benchmark, we compare BatVI with other methods. We show that BatVI can predict more correct HBV integrations and produce less false positives in the shortest amount of time. In summary, BatVI is fast, sensitive and accurate.

## Methods

The input of BatVI consists of a database of viruses, a human reference genome and the raw NGS reads (or the SAM/BAM alignments of these read to the human genome). BatVI has three stages. First, it identifies a set of chimeric read pairs that map both to human and virus genomes. Next, the chimeric reads that co-localize in the human genome are clustered. Finally, integration sites are extracted from these clusters. These stages are described in detail below.

### Identifying Chimeric Reads

BatVI can start with either the raw reads or a SAM/BAM file containing the read alignments. If the SAM/BAM file is given, we extract read pairs with soft-clips and those with at least one read unmapped. Otherwise, the whole set of raw reads is taken as the input. These reads are checked for the presence of a virus. This is done by checking if some *k*-mer from the reads can be mapped to the virus database with at most *r* mismatches. To be sensitive, we set *k*=18 and *r*=1 by default. Once we check one *k*-mer in the read, we shift by *s* positions (*s*=5, by default) and check another *k*-mer on the read. The alignment of *k*-mers is done using BatMis [16] algorithm, which is a BWT-based algorithm that can report all hits. We retain read pairs with at least one *k*-mer aligned on the virus database. Such a set of read pairs is denoted as the set *X*_1_. After this initial screening, the second step performs a more thorough examination for a viral segment in the reads in *X*_1_ by aligning them to the virus database using BLAST. All the read pairs that do not have a mapping by BLAST are discarded and the remaining read pairs are stored in the set *X*_2_. *X*_2_ is a set of read pairs that possibly originated from the virus. Finally we check if the read pairs in *X*_2_ can be mapped to the human genome by BLAST. If a read or its mate has a hit in human genome by BLAST, it is stored in the set *X*_*chimera*_.

**Fig. 1:**
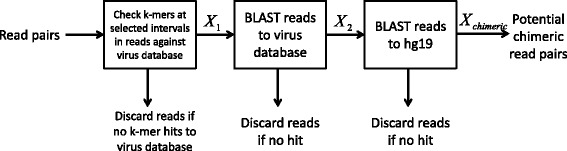
A pipeline to identify potential chimeric reads

Although BLAST is accurate, it is several times slower compared to NGS aligners. Hence, this pipeline is carefully adjusted to minimize the usage of BLAST. We use it sparingly by first removing a set of reads unlikely to be chimeras, and then using BLAST with a small virus database, and finally with the reference genome. Figure 1 shows the complete pipeline.

### Clustering reads

Let *d* and *σ* be the mean and standard deviations of the insert size of the read pairs. For each read *R*_*i*_∈*X*_*chimera*_ that is mapped to human, BLAST will report a set of locations ${L^{i}_{1}},{L^{i}_{2}}, \ldots, {L^{i}_{j}}$ with expect values ${e^{i}_{1}},{e^{i}_{2}}, \ldots, {e^{i}_{j}}$. The hit ${L_{l}^{i}}$ is called a rank-*N* hit if there are *N* hits whose expect values are smaller than or equal to ${e_{l}^{i}}$. (The rank will be used in Section “Identify integration breakpoints”). If there are multiple hits with the smallest expect value, we retain all hits with the smallest expected value. If there is exactly one hit with the smallest expect value, we also retain hits with the second smallest expect value. (We retain the second best hits since the unique best hit may be noise. Keeping the second best hits increases our chance of finding the correct hit. This trick is also used in [17]).

Among all retained hits $(R_{i}, {L_{l}^{i}})$, we put the hits into the same cluster if they are near each other. Precisely, we sort the hits for each chromosome in the ascending order of their location. Then we traverse this sorted list chromosome by chromosome, from top to bottom. Clusters are formed during this traversal by adding two consecutive hits $(R_{i}, {L^{i}_{p}})$ and $(R_{j}, {L^{j}_{q}})$ to the same cluster if $|{L^{i}_{p}}-{L^{j}_{q}}|<d+2\sigma $.

**Fig. 2:**
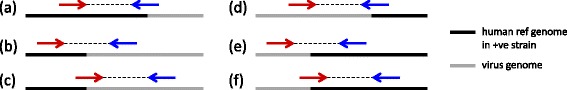
This figure illustrates the orientation of the chimera reads when they map on the human genome. For all examples, we orient the human-virus integration fragments such that the human reference is in +ve strain. **a**–**c** illustrate cases where human is on the 5’ side of virus. In such cases, for each read *R*_*i*_ aligned on the human genome, we have either the whole read *R*_*i*_ or its prefix aligns on the +ve strand of the human genome or only the prefix of *R*_*i*_ aligns on the -ve strand of the human genome. **d**–**f** illustrate cases where human is on the 3’ side of virus. In such cases, for every read *R*_*i*_ aligned on the human, we have either *R*_*i*_ or its suffix aligns on the -ve strain of the human genome or only the suffix of *R*_*i*_ aligns on the +ve strain of the human genome

The next step is to partition the aligned reads in every read cluster *C*_*i*_ into two sub-clusters $C^{+}_{i}$ and $C^{-}_{i}$ such that $C^{+}_{i}$ contains all reads that align on human-virus integrations where human is on the 5’ side of the virus. The classification can be done based on the alignment orientations of the reads on the human genome as illustrated in Fig. 2. Precisely, for every $(R_{a},{L^{a}_{j}})\in C_{i}$, if the prefix of *R*_*a*_ aligns on the +ve strand or the suffix of *R*_*a*_ aligns on the -ve strand of human genome, we add $(R_{a}, {L^{a}_{j}})$ to the sub-cluster $C^{+}_{i}$; otherwise, it is added to $C^{-}_{i}$.

### Extract integration sites

**Fig. 3:**
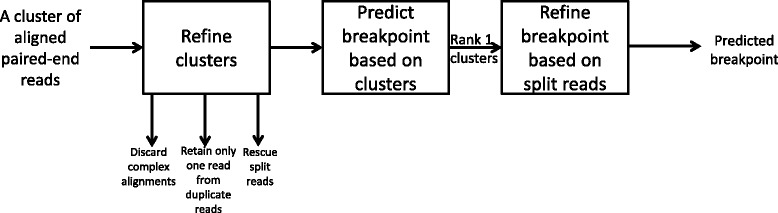
The work flow showing how clusters are refined and breakpoints are predicted

After clustering the reads, we follow three steps to identify the possible integrations (see Fig. 3). First, we refine the clusters to remove noisy and duplicate reads. Second, we identify a possible breakpoint from the clusters. Finally, if split reads are present, we use them to refine the breakpoints. These steps are detailed below.

#### Refining the Clusters

The clusters are refined by 1) removing noisy and complex reads, 2) filtering duplicate reads and 3) rescuing split-reads. We describe the details of the three steps below.

**Fig. 4:**
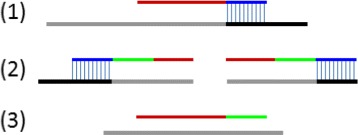
The figure shows how the human segment of a read may be unaligned by BLAST. The black and gray lines indicate the human and viral reference genomes, respectively. The red segments are sequences originating from viral genome and the blue segments originate from the human genome. The green segment indicates a random sequence and the blue vertical lines indicate places where reference and the human segment match. In (*1*), although the human segment matches the reference, it is too short to be detected by BLAST. In (*2*), a random sequence is present in the integration and the human segment present is too short to be detected by BLAST. In (*3*), their is no human segment at all. This may be due to an insertion or due to a misalignment of the sequence. We will attempt to rescue reads in cases (1) and (2) through local alignment

(1) BatVI assumes that each single ended read is composed of at most one segment from human and at most one segment from virus. If there are other complex cases, BatVI will discard these reads. (For example, if there are reads with human segment between two viral segments in one read, we discard such read pairs.) If both ends of a read pair are mapped to the human genome, we check to see if they are correctly oriented and whether they map within the same chromosome. If they are not, the reads are discarded.

(2) Due to the low number of viral integrations in a cell population, it is highly unlikely that the datasets contain identically mapped read pairs unless the sequencing was targeted. Therefore, whenever there are identical hits (in terms of alignment), we provide the option to retain only one copy and remove the other reads from the cluster.

(3) Some reads align partially to the human genome or to the virus. BLAST fails to align the remaining portion of such reads due to several reasons. The first reason is the limits of sensitivity of BLAST, where sequences of length 25 bp is required for an alignment. The second reason is due to a short random sequence inserted within the viral-human integration site. The third reason is that the alignment may be incorrect. (See Fig. 4.) We try to rescue the alignment of the split reads for the first two cases.

If one side of a read aligns to the virus by BLAST, and there is at least 10 contiguous bases unaligned, we attempt to rescue this read as a split-read. The mate of such a read must be aligned to human. If the mate is not a split-read, we may be able to rescue the split-read as follows. We extract the sequence flanking the mate. The size of the flanking region is set as *d*+2∗*σ*. If the split read is real, the unaligned portion of the read should align within this extracted flanking region. We use a fast SIMD-based implementation of the Smith-Waterman algorithm to check if this is the case. If no such pattern is found, we discard the read. Otherwise, the read is updated as a true split read.

#### Identify integration breakpoints

**Fig. 5:**
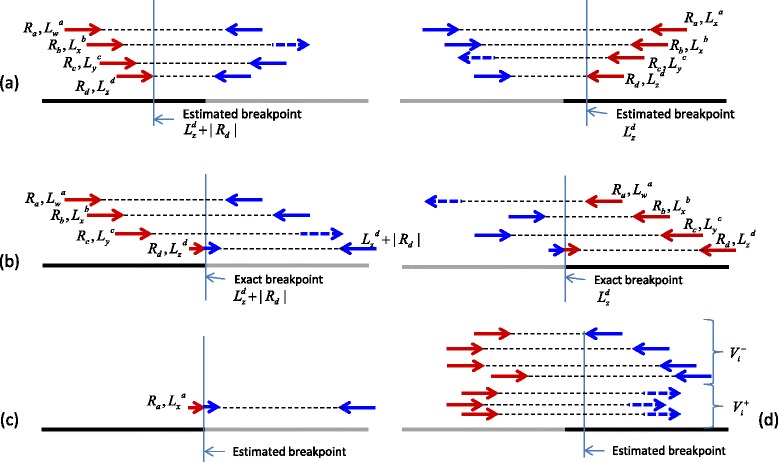
The figure shows how the breakpoints are estimated from a cluster of reads. The red segments of a read aligns to human genome (shown as a *black* line), and the blue segments belong to the viral genome (shown as a *gray* line). The solid arrows show properly aligned reads and dashed arrows indicate reads that are aligned incorrectly. For a read cluster $C^{+}_{i}$ (or $C^{-}_{i}$) we take the 3’-most(5’-most) aligned position of the read cluster as the estimated human breakpoint. In (**a**), there is no read passing through the actual breakpoint so the estimation can be off to the 3’ side (or 5’ side). This can be as much as the maximum insert size span of the library. However, if there is a split read *R*_*d*_ (**b**), the exact human breakpoint can be recovered. To find the viral co-ordinate of the integration following procedure can be used. If a split read is available close to the estimated human breakpoint, the exact viral breakpoint can be found out **c**. Otherwise, the viral mappings of the cluster $C^{+}_{i}$ (or $C^{-}_{i}$) will be further sub-divided into two clusters based on the strand of the mapping. The cluster containing the largest number of reads will be considered as correct. Then, the viral breakpoints can be estimated using similar method as that for the human breakpoints **d**

For each cluster $C_{i}^{+}$ (or $C^{-}_{i}$), we estimate the integration breakpoints on both human and virus as follows. To estimate the human integration breakpoint for a cluster $C^{+}_{i}$, we report $\max \left \{len(R_{a})+{L^{a}_{j}}|(R_{a},{L^{a}_{j}})\in C^{+}_{i}\right \}$. Similarly, the human integration breakpoint for a cluster $C_{i}^{-}$ is estimated as $\min \left \{{L^{a}_{j}} \mid (R_{a}, {L^{a}_{j}}) \in C^{-}_{i} \right \}$ (see Fig. 5 a). In the case of the clusters containing a split read with a mapping for the human segment, we can estimate the exact breakpoint (see Fig. 5 b).

**Fig. 6:**
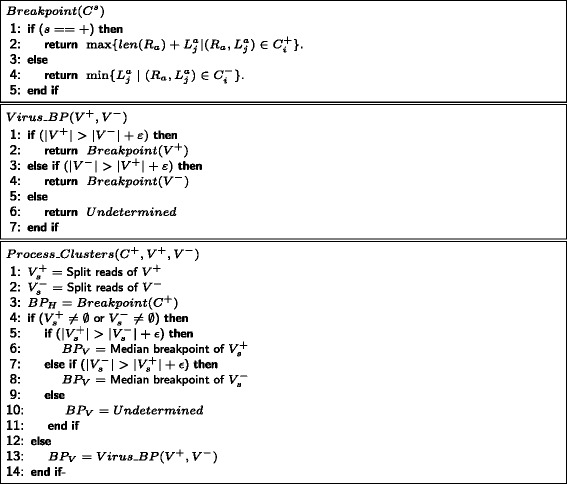
Algorithm showing how the breakpoints are found for $C^{+}_{i}$ clusters

To estimate the virus integration breakpoint, the reads in $C^{+}_{i}$ (or $C^{-}_{i}$) and their mates having a viral mapping are considered. Similar to the detection of the human integration, these reads are clustered into two groups: Those mapping to the positive strand of the viral genome ($V^{+}_{i}$), and those mapping to the reverse strand of the viral genome ($V^{-}_{i}$). If the alignments are accurate, one of these clusters should be empty. However, noisy mappings might make both clusters non-empty. When one cluster contains at least *ε* reads more than the other cluster (*ε*=2 by default), we assume that cluster to be the correct one. Otherwise, the viral breakpoint is not reported. The orientation of the viral segment can be determined based on the strand of the reads in the viral cluster and the orientation of the human breakpoint. For example, if the human breakpoint is deduced from a cluster $C^{+}_{i}$ and the viral segment contains reverse strand reads, the 5’ most position of the viral segment is closest to the breakpoint (see Fig. 5 d). If the viral reads are from the +ve strand, the 3’ most position of the viral cluster is the closest to the breakpoint (see the procedure *V* *i* *r* *u* *s*_*B* *P* in Fig. 6). If there are reads spanning across a breakpoint as shown in Fig. 5 c, the exact breakpoint can be found.

The clusters generated in the clustering step contain numerous random clusters. We need to filter them out. For each cluster $C^{+}_{i}$, its median rank is defined as the median of the ranks of ${L^{a}_{j}}$ for all $(R_{a},{L^{a}_{j}})\in C^{+}_{i}$. (Note: the rank of ${L^{a}_{j}}$ is defined in Section “Clustering reads”). The median rank for $C^{-}_{i}$ is similarly defined. We only retain clusters with a median rank of 1.

#### Refining the Breakpoints

The predicted breakpoint of each cluster might not be accurate due to a noisy mapping affecting the calculation of the end points of $V^{+}_{i}$ clusters and $V^{-}_{i}$ clusters. It is possible that virus integrations in different cells are different, but are close to each other. If the final clusters contain split reads, we can use them to refine the predicted breakpoints under such circumstances.

The split reads can predict the integration sites to a high degree of accuracy. However, BLAST may align split reads to the virus and human genome off by several bases. From all the split reads, we find the human and viral alignments and take their median value *M* to be the exact breakpoint. We report this median as the actual break point if one of the read clusters contain at least *ε* split reads more than the other (see the procedure *P* *r* *o* *c* *e* *s* *s*_*C* *l* *u* *s* *t* *e* *r* *s* in Fig. 6). Note that in some cases read clusters may not have a median rank 1 when there are many reads mapping to different locations with the same expect values. In such cases or when split reads are not present, breakpoints can be estimated by local assembly. First, we identify aligned reads $(R_{a}, {L^{a}_{j}})$ and $(R_{b}, L^{b}_{j'})$ in a cluster such that *R*_*a*_ and *R*_*b*_ overlap by at least 30 bp with similarity >75*%*. Next, we pile-up all these read pairs to generate their consensus sequence. These consensus sequences are mapped to the reference genome with BLAST. If there exists an alignment with a unique smallest expect value, the unaligned portion of this consensus sequence is mapped to the virus database using BLAST. If there is a hit, the integration is reported. If there are multiple hits to the human genome with the same expect value, multiple breakpoints are predicted and these breakpoints are marked as ambiguous breakpoints.

## Results

This section studies the performance of BatVI, VirusFinder [8, 9] and VirusSeq [6]. ViralFusionSeq [7] is not included in the comparison since it cannot finish running within two weeks in our experiments. The details of the simulation and real-data experiments are given below.

### Generation of simulated data

Using the simulator program in VirusFusionSeq [7], an infected genome is simulated where chromosomes 1–4 are infected by four different HBV strains. Then, all integrated viral regions, along with their two 500 bp flanking human regions were extracted. Next, using the default parameters of the Mason simulator [18], a 20X coverage dataset of these extracted regions were generated. Altogether, 1762 integration sites were generated in this simulation.

From the simulated dataset, we downsampled it and created two additional simulated datasets that contain 50% and 25% of the original reads.

### Integration detection in simulated data

This section compares the performance of BatVI, VirusFinder 2 [9] and VirusSeq [6] on the simulated datasets generated in the previous section. In this comparison, a prediction was considered to be correct if it was within 300 bases from the simulated breakpoints of some simulated viral integration and with the correct orientation. VirusFinder 2 reports two types of integrations designated as high-confident and low-confident. To increase its sensitivity, we pool these two types of integrations together.

**Fig. 7:**
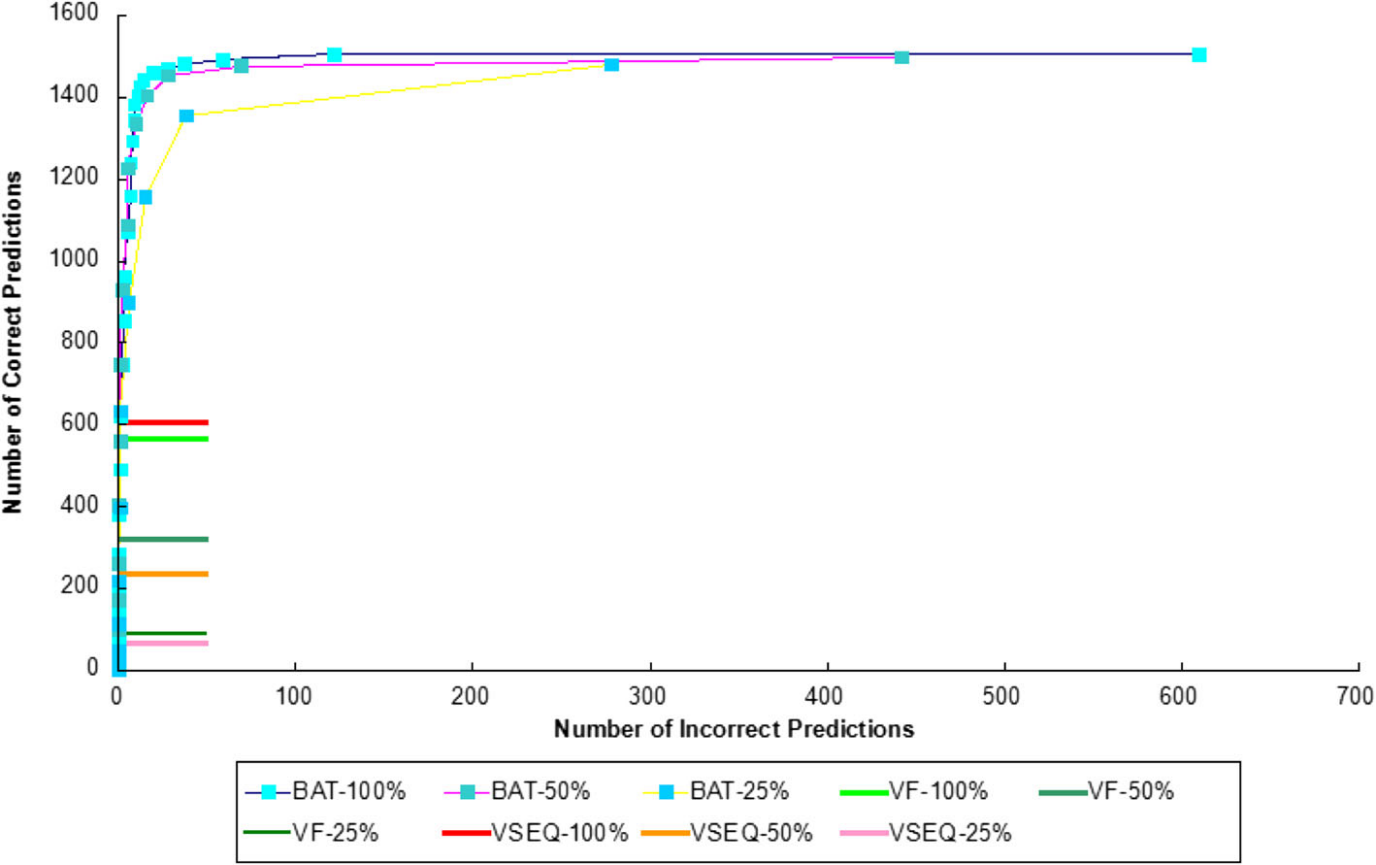
The change of false positives and true positives with the number of reads used to predict an integration with BatVI. The x-axis is log-scaled. The plots for the comparisons except BatVI are shown as straight lines for clarity, but they are in fact points with x values not exceeding 0

Figure 7 shows the ROC of BatVI (i.e. the number of correct predictions versus the number of incorrect predictions thresholded by the minimum number of supporting reads), along with the predictions of VirusFinder 2 [9] and VirusSeq [6] under different sequencing depths.

Without downsampling, VirusFinder 2 and VirusSeq identify 32% and 35% of the true integrations with about 1% and 0.1% false positives respectively. For BatVI, if we take the viral integrations supported by at least one read, BatVI identifies 85% of the true positives, with 28% false positives. However, if we select a more stringent cutoff for BatVI by increasing the number of reads supporting the predictions, the false positive rate rapidly goes down (see Fig. 7). BatVI can predict more than twice true positives compared to other programs under 1*%* false positive rate.

In addition, as the sequencing depth is reduced, the number of predictions by the other programs decrease very rapidly. For VirusFinder 2, 42% and 84% of predictions are lost at 50% and 25% sampling rates respectively. For VirusSeq, 60% and 95% of the predictions are lost at 50% and 25% sampling rates respectively. However, BatVI is more robust. If we take the number of predictions with read count cutoff chosen so that the false positive rate is kept at less than 1%, BatVI loses only 7% and 38% of the integrations at 50% and 25% sampling rates respectively.

**Fig. 8:**
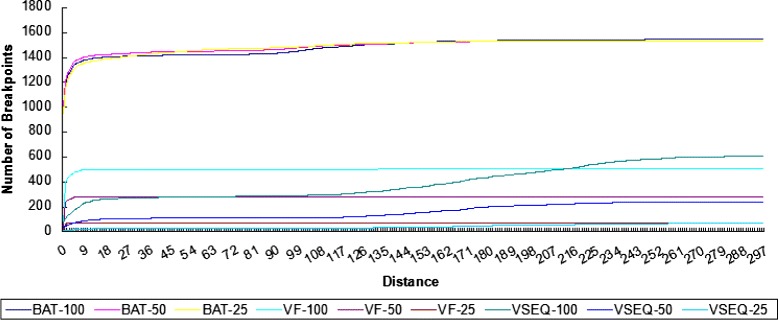
The graph shows the distribution of the distance between the exact breakpoint and the predicted breakpoint for different programs

Next, we study the distance between the actual breakpoints and the predicted breakpoints. Figure 8 shows the result. The predictions by BatVI and VirusFinder 2 are very close to the actual breakpoint most of the time. However, more than half of the breakpoints reported by VirusSeq can be as far as 100 bp away from the actual breakpoints.

### Performance on real data

This section compares the performance of BatVI, VirusFinder and VirusSeq using real datasets. We ran the tests in the high-performance cluster at Genome Institute of Singapore, allocating 32GB of memory and ten threads with a time limit set at 14 days.

In [8], VirusFinder 1[8] and VirusSeq [6] were compared using two WGS libraries 198T and 268T from [19]. In the first experiment, we run BatVI on these two datasets. BatVI finished the virus integration calling in several hours. VirusFinder took one week to process the library 268T and it crashed on the library 198T. VirusSeq did not finish even after two weeks. In fact, [8] also failed to process 198T and 268T using VirusSeq within the allowed time.

Table 1 lists all HBV integrations reported by the original paper [19] and predicted by BatVI and VirusFinder 1. (Since we fail to run VirusFinder 1, the integration list of VirusFinder 1 is extracted from the original paper [8]). VirusFinder 1 reported 2 and 3 integrations for libraries 198T and 268T respectively. BatVI reported the superset of HBV integrations reported by VirusFinder 1. If we consider the integrations supported by at least three reads, BatVI reported 6 integrations for each library. The extra integrations reported by BatVI were also reported in the original study within a deviation of 30 bp. (Three integrations reported in the library 268T appear within 300 bp to an integration reported in the original study and may refer to the same breakpoint).

**Table 1:** Comparison of integrations reported by BatVI, VirusFinder 1 and the original paper for the libraries 268T and 198T

	BatVI	Original	VirusFinder 1	Library
chr10	125472276	125472277	-	198T
chr10	131711533	131711538	-	198T
chr10	131726472	131726472	-	198T
chr19	30297359	30297360	-	268T
chr19	30298788	30298788	30298787	268T
chr5	1269391	1269361	1269387	198T
chr5	1269406	1269406	1269405	198T
chr5	1292392	1292393	1292391	268T
chr5	1292073	-	-	268T
chr5	1292329	-	-	268T
chr5	1292404	1292404	1292403	268T
chr8	82390663	82390663	-	198T
chr3	-	191648206	-	198T

The second experiment studies the performance of VirusFinder 2 [9], VirusSeq [6] and BatVI on 7 samples in [19] that have viral integrations reported by HIVID [15]. (HIVID is based on capture sequencing. It has high sequencing depth. Here, we treat it as a golden benchmark dataset). Since VirusFinder 2 and VirusSeq were slow to run, we downsampled the selected libraries and tested the performance of different methods on them.

Each selected sample has two libraries: The first library contained reads having an insert size of 170 bp while the other library contained reads having an insert size of 800 bp. We downsampled them as follows. First, we align these reads on the human genome using BWA [20]. Paired-end reads having soft-clips or with at least one side unmapped were extracted. These paired-end read are more likely to be originated from viruses or their integrations in the human genome. In addition to these paired-end reads, one million random paired-end reads were extracted from each library. In total, 14 datasets are obtained (see Table 2 for the number of paired-end reads selected in these datasets). Then each dataset was processed using BatVI, VirusFinder 2 and VirusSeq. For BatVI, we report integrations supported by at least four paired-end reads. For the 170 bp and 800 bp datasets, two integrations were considered to be the same if they are within 200 bp and 800 bp away from each other respectively.

**Table 2:** Table showing the user time in seconds taken for each program to process a set of libraries

Library	VirusSeq	Virusfinder 2	BatVI
145T.170	139063.09	10681.89	1068.59
145T.800	142839.84	9708.24	1818.19
174T.170	138189.30	8404.97	347.36
174T.800	174110.80	11255.71	895.41
182T.170	138965.89	10423.38	397.17
182T.800	143127.62	12803.67	321.79
186T.170	119893.79	10519.19	221.69
186T.800	121806.51	11444.95	220.89
23T.170	118660.10	10662.88	457.60
23T.800	116794.80	11226.10	517.36
266T.170	118169.59	10419.90	344.68
266T.800	96608.79	9852.84	240.10
32T.170	178890.78	9734.77	271.37
32T.800	139353.66	8969.77	231.51
32T.800	139353.66	8969.77	231.51

The libraries are sub-samplings of real life data. The libraries with suffix 170 have an average insert size of 170 bp while those with the suffix 800 have an average insert size of 800 bp

**Fig. 9:**
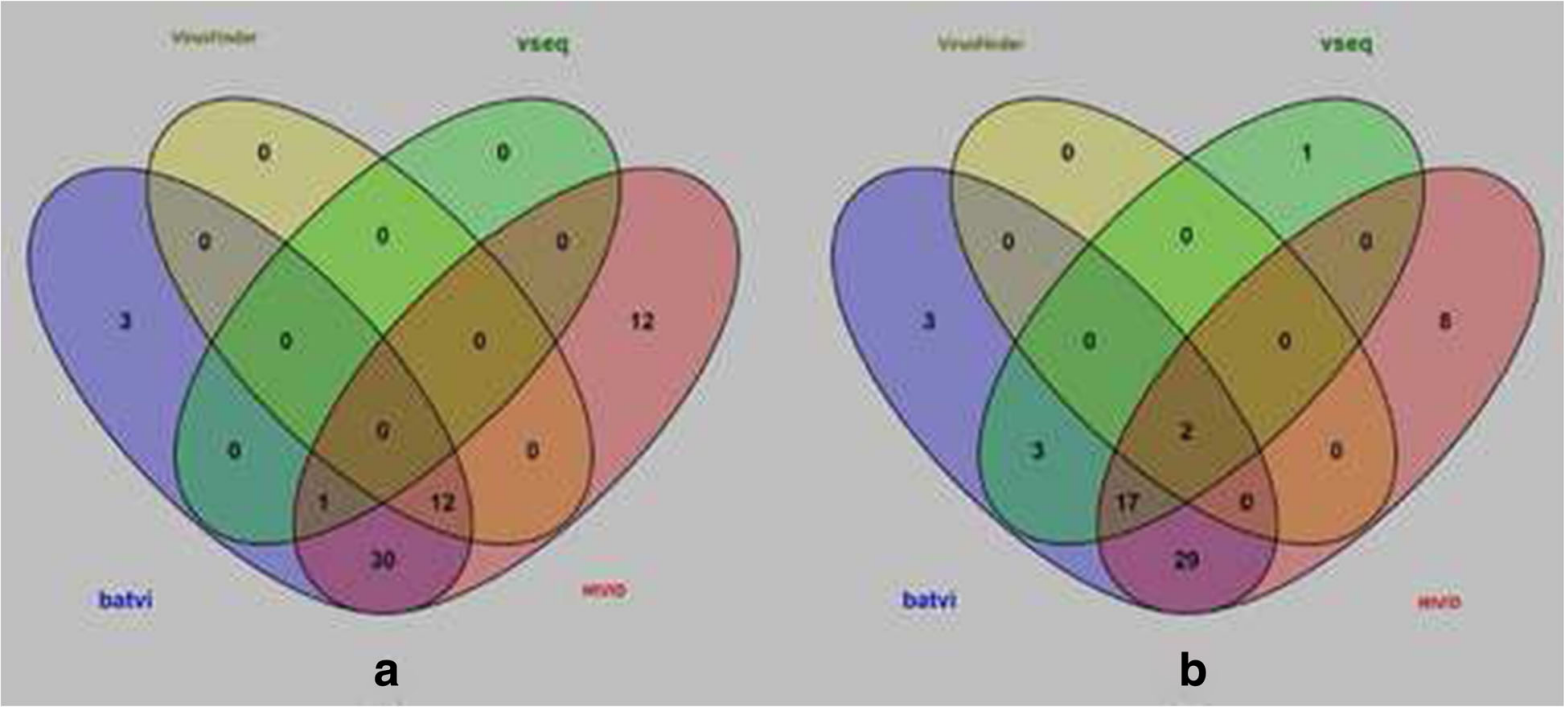
The venn diagrams for the HBV integrations reported by BatVI, VirusFinder 2, VirusSeq and HIVID. **a** is the Venn diagram for 7 samples with insert size 170 bp. **b** is the Venn diagram for the same 7 samples with insert size 800 bp

Figure 9 shows the intersection of these results. BatVI result is almost a superset of other programs. VirusFinder 2 and VirusSeq are the second best in terms of the number of predictions on 170 bp and 800 bp read classes respectively. If we take HIVID results as the correct predictions, for the 170 bp reads, 93% of BatVI predictions are validated and it reports nearly twice more validated predictions compared to VirusFinder 2. For the 800 bp dataset, 88% of BatVI predictions are validated, while it again reports nearly twice more validated predictions compared to VirusSeq. Also, all the validated hits of the other programs were reported by BatVI. Another observation is that VirusSeq reports just a single integration in the 170 bp dataset while VirusFinder 2 reports only 2 integrations in the 800 bp dataset (See Table 3). This indicates that VirusSeq cannot work for paired-end reads with small insert size while VirusFinder 2 cannot work for paired-end reads with large insert size. (Note: It is expected that VirusFinder 2 cannot work for large insert size since it is based on CREST and CREST cannot work well for large insert size).

**Table 3:** Comparison of viral integrations reported by VirusFinder 2, VirusSeq and BatVI on 14 downsampled libraries. The library suffixes indicate the expected insert size

	VirusSeq	Vfinder	BatVI
145T.170	0	3	11
145T.800	6	0	15
174T.170	0	0	1
174T.800	1	1	6
182T.170	0	1	3
182T.800	3	0	3
186T.170	0	0	2
186T.800	3	0	2
23T.170	0	6	15
23T.800	8	1	15
266T.170	0	2	10
266T.800	0	0	8
32T.170	1	0	4
32T.800	2	0	5

### Reanalysis of data from Sung et. al.

We ran BatVI on all 87 samples of liver cancer data from [19]. The original analysis reported 399 integration sites and our new analysis revealed 812 integrations supported by more than one read. BatVI detects 341 of the original breakpoints (integrations are considered to be the same if they occured within 800 bp away from each other). To get a better idea about the predictions, we compared the predictions for HIVID, BatVI and the original analysis in the samples where HIVID validation is available. Out of the 246 HIVID predictions, original analysis found 115 (47*%*) HIVID validated integrations while BatVI found 133 (54*%*) HIVID validated integrations. This shows that BatVI is more sensitive in detecting these confident integrations.

**Fig. 10:**
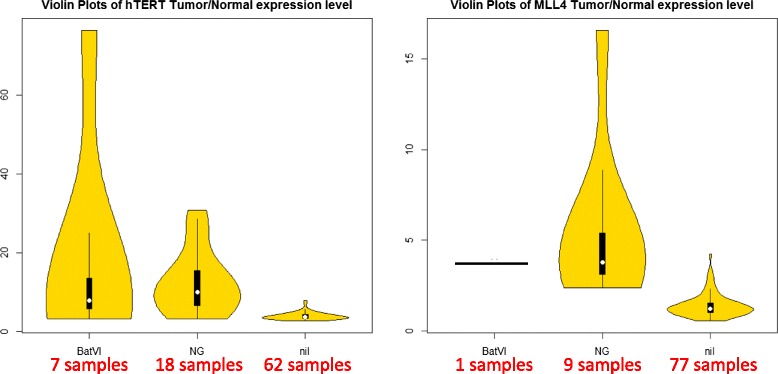
The violin plots on the left and on the right show the tumor/normal expression ratios of TERT and MLL4, respectively. For each plot, the 87 samples are partitioned into three violin plots. The second violin plot (NG) corresponds to the original samples where the HBV integrations were detected. The first violin plot (BatVI) corresponds to the extra samples where the HBV integrations were detected. The third violin plot (nil) corresponds to the samples with no HBV integration detected

The original analysis [19] identified 31 out of 87 samples containing recurrent HBV integrations with TERT, MLL4 and CCNE1 oncogenes. BatVI was able to identify recurrent HBV integrations in all these samples. Furthermore, it identified 7 more samples with HBV-TERT integration and an extra sample with the HBV-MLL4 integration. We know that HBV integration will up-regulate the expression of TERT and MLL4. When we analyze the gene expression data for these 8 extra samples, we see that TERT and MLL4 expressions are higher in tumor samples compared to the normal samples, relative to the samples where no such integration were found (see Fig. 10). This shows that the additional HBV integrations predicted by BatVI are likely to be real. Our analysis indicates that the original study gave 8 false negatives (i.e the original analysis was able to find HBV integrations to this listed oncogenes in 39% of the samples while BatVI can detect them in 49% of the samples). Note that this difference may have clinically significant consequences since the false negatives may lead to incorrect treatment for the patients.

### Speed comparison

This section performs the efficiency comparison of VirusFinder 2, VirusSeq and BatVI. For the speed comparison, we used the 14 downsampled datasets as stated in Table 2. Table 2 reports the real times taken by each program in seconds.

The table shows that the speeds of the programs differ by at least an order of magnitude in general. VirusSeq is very slow compared to the other programs. It will be challenging to perform viral integration studied on large datasets using VirusSeq. VirusFinder 2 is more efficient but it is clearly much slower than BatVI. In practice, we observe that finding viral integrations with BatVI can be more efficient than aligning the reads to a human genome using BWA [20].

## Discussion

BatVI uses two criteria for judging the quality of a viral integration: the rank and the number of reads in a cluster. Rank 1 clusters of BatVI are the unambiguous viral integrations. Currently, BatVI only reports rank 1 clusters. For the remaining clusters, they might fall on repeat regions and we currently store them separately. Users can inspect these remaining clusters if they want.

For read count, a cluster with a higher read count has higher confident. However, we do not impose a cutoff on reporting integrations based on the read count. The reason is that the read count depends on the depth of sequencing and the frequency of cells with integrations. Therefore, the correct cutoff for the read count depends on the experimental setting. The user can adjust the cutoff based on their knowledge on the datasets.

## Conclusion

Discovering viral integrations using NGS data has become important especially for disease samples that have strong association with viral infections. However our experiments show that existing methods are slow and are not sensitive enough. We developed an algorithm BatVI, and show that it is much faster than existing programs while being more sensitive. Also, it does not show any bias towards the insert size of the libraries in contrast to the other methods compared. We believe that BatVI will be a useful tool for studying viral integrations in future.
